# The EU Interreg Project “ADRINET”: Assessment of Well-Known and Emerging Pollutants in Seafood and Their Potential Effects for Food Safety

**DOI:** 10.3390/foods13081235

**Published:** 2024-04-17

**Authors:** Elisabetta Bonerba, Fatmira Shehu, Annamaria Pandiscia, Patrizio Lorusso, Alessio Manfredi, Aleksandra Huter, Giuseppina M. Tantillo, Sara Panseri, Maria Nobile, Valentina Terio

**Affiliations:** 1 Department of Veterinary Medicine, University of Bari Aldo Moro, Provincial Road to Casamassima km 3, 70010 Valenzano, Italy; elisabetta.bonerba@uniba.it (E.B.); patrizio.lorusso@uniba.it (P.L.); alessio.manfredi@uniba.it (A.M.); valentina.terio@uniba.it (V.T.); 2 Department of Veterinary Public Health, Faculty of Veterinary Medicine, Agricultural University of Tirana, 1025 Tirana, Albania; s_fatmira@yahoo.it; 3 Institute of Marine Biology, University of Montenegro, 85330 Kotor, Montenegro; aleksrhn@gmail.com; 4 Department of Interdisciplinary Medicine, University of Bari, Place Giulio Cesare 11, 70124 Bari, Italy; m.tantillo@tim.it; 5 Department of Veterinary Medicine and Animal Science, University of Milan, Via dell’Università 6, 26900 Lodi, Italy; sara.panseri@unimi.it (S.P.); maria.nobile1@unimi.it (M.N.)

**Keywords:** food safety, chemical contaminants, microplastics, antibiotics

## Abstract

Anthropogenic activities lead to the spread of chemicals and biological materials, including plastic waste, toxic metals, and pharmaceuticals, of which the impact on the Mediterranean Sea is of high concern. In this context, the EU Interreg Italy-Albania-Montenegro Project “ADRINET (Adriatic Network for Marine Ecosystem) _244” (2018–2020) arises. It aims to carry out biomonitoring campaigns in the main commercial interest of fish and cephalopod species, such as *Sparus aurata*, *Dicentrarchus labrax*, *Sepia* spp., and *Loligo* spp. sampled in three different subregions of the Mediterranean Sea. The presence of the main environmental contaminants, such as cadmium, microplastics, and antibiotics was investigated in these seafood samples. Contamination by cadmium and antibiotics in the seafood investigated in our study was negligible. However, a high value of microplastics was detected in the stomach and gut of *Sparus aurata* and *Dicentrarchus labrax*. Overall, even though the presence of microplastics needs to be investigated by further studies, the results confirmed that the environmental conditions of the three bays investigated by the ADRINET project partners (Italy, Albania, Montenegro) are positive and not affected by intensive anthropogenic activity.

## 1. Introduction

Scientific interest in the monitoring of well-known and emerging pollutants from the marine environment has increased in recent decades. Specifically, the environment of the Mediterranean sea has been investigated as a unique biodiversity hotspot of marine fauna and flora. However, it is also one of the most vulnerable ecosystems due to its hydro geographical characteristic and highly polluting industrial and anthropogenic activities [1]. Overall, the latter are a complex and ever-changing mixture of chemicals and biological materials, including plastic waste, petroleum-based pollutants, toxic metals, manufactured chemicals, pharmaceuticals, pesticides, phosphorus, fertilizer, and sewage. Their impact on deep-sea ecosystems has been to be proven significant within the Mediterranean Sea. They also pose a serious threat to human health through entry into the food chain since the most common exposure to these contaminants for humans is fish and seafood consumption [1,2,3,4].

According to the European Environment Agency (EEA) report, 87% of the pollution of the Mediterranean Sea has been caused by toxic metals, industrial chemicals, and plastic waste [5]. Heavy metals represent one of the most prevalent and toxic elements commonly found in aquatic environments. Among all, cadmium (Cd) is considered the major anthropogenic contaminant in coastal and marine environments worldwide [6]. It poses a serious hazard to living organisms and natural ecosystems due to its toxicity, persistence, and bioaccumulation characteristics [7]. In fishes, Cd determines disfunctions in growth and development, reproductive processes, osmoregulation, morphological structures, histology, stress tolerance, and the endocrine system [6]. In humans, Cd leads to cancerogenic effects affecting multiple organ functions via biomagnifications through food chains [8].

Among the emerging pollutants, plastics and antibiotics are considered one of the contaminants of greatest concern [9,10].

Due to the massive production and use of plastics in both industry and daily life, plastic wastes are ubiquitously present in various environments, forming an emerging pollution phenomenon named plastic pollution [11]. Microplastics (MPs) are pieces of plastics measuring about 5 mm that have accumulated and now persist in natural aquatic ecosystems [12,13]. They become harmful to marine life once ingested by fish. In fact, they can cause various types of damage, such as a decrease in reproductive capacity and/or an increase in mortality [14]. In addition, due to their large specific surface area and hydrophobicity, MPs may adsorb other environmental pollutants, such as several antibiotics, through Van der Waals forces, pore filling, and electrostatic interactions contributing to the spread of public concerns of antibiotic resistance [15,16,17].

Despite current scientific data on ocean pollution levels, there are still large gaps in knowledge about sources and levels of pollution in many areas of the sea, high-risk populations, the extent of human exposure, and health effects [18]. Nevertheless, it is well known that contaminants accumulated in seawater will bioaccumulate in fish reaching the human body through the food chain with serious risks for food safety and human health [18].

In this context, the EU Interreg Italy-Albania- Montenegro Project “ADRINET (Adriatic Network for Marine Ecosystem) _244” arises, which started in 2018 and ended in 2020 [19,20].

The ADRINET Project gathered scientists, experts, and fishermen from Italian, Albanian, and Montenegro Partners, with the aim of improving a joint coastal management system and creating governance plans to preserve biodiversity and coastal ecosystems inside the Programme Areas by involving local communities strongly characterized and influenced by fishing activity.

The selected sub-regions share the same issues in terms of pollution, the over-exploitation of fish stocks, illegal fisheries, and “ghost fishing”. The project includes investments in technology, to map fishing routes and fishing equipment (gears, traps, nets) to help their traceability over the long-term period, to monitor sea pollution, and provides services, scientific support, and skills for fisheries professionals and consumers, to make fish consumption safer and compliant with EU rules and guidelines.

ADRINET is part of the sixth European Community Environmental Action Program 2002–2012 (6th EAP), which identifies four environmental areas for priority actions: “Climate change”, “Nature and biodiversity”, “Environment, health and quality of life”, and “Resources and natural waste”.

The project aims to improve a common coastal management system to preserve the biodiversity and the marine ecosystems of the sub-regions involved.

The ADRINET project has analyzed data relating to the current situation of the marine ecosystem of the fishing area of the Castro Bay, located in the northwestern Ionian Sea (GSA19) on the border of the southern Adriatic Sea (GSA18), of the Vlore Bay (GSA18) and of the Boka Kotorska Bay (GSA18), connected to the issues of greatest impact, such as fishing techniques and pollution, for the assessment and management of risks related to the maintenance of the “fragile” balance of the marine ecosystems.

In particular, to assess the impact of fishing, ADRINET has included the analysis of the following:

- the type of fishing gears used and the practice (higher impact by dragged gear and particularly dredges);
- the geographic location of the activity (and its intensity);
- the type of habitat, its status, and its environment, together with the marine species and communities.

As concerns this issue, an environmental risk management plan has been planned through the scientific activity of the three Universities involved (University of Bari Aldo Moro, Department of Veterinary Medicine (Lead Partner), Univerzitet Crne Gore/Institut za biologiju mora—University of Montenegro/Institute of Marine Biology, Universiteti Bujqesor i Tiranes—Agriculture University of Tirana).

This scientific evaluation continued even after the end of the project, until 2023, with the aim of providing harmonized results within the three research groups involved.

Therefore, the present study aims to carry out biomonitoring campaigns for the main fish and cephalopod species of commercial interest from the Adriatic-Ionian region of the Mediterranean Sea, such as *Sparus aurata*, *Dicentrarchus labrax*, *Sepia* spp., and *Loligo* spp.

This study investigates the presence of the main environmental contaminants, such as cadmium, microplastics, and antibiotics, in these fishes and cephalopods, sampled in the three different fishing areas indicated above. Moreover, the ADRINET project focuses on the delivery of a scientific methodology to assess, monitor, and control sea water conditions and human-related risks regarding seafood in identified areas, producing a comprehensive set of tools (e.g., ERMP, Handbook on joint management of pollution-related risks, Memorandum of Understanding for coordination on sustainable use of marine ecosystems) aimed at enhancing environmental risk management by sharing knowledge and policies. This result will be linked to environmental protection, as well as to the fostering of a sustainable Blue Economy and, finally, to the management of food safety.

## 2. Material and Methods

### 2.1. Sampling

Based on the main fish species present in every bay, during the years 2018–2020, the sampling was carried out as detailed below: mantles and glands from 80 *Sepia* spp. and 80 *Loligo* spp. were collected for cadmium analysis; stomachs and guts from 90 *Sparus aurata* and 70 of *Dicentrarchus labrax* were sampled for microplastics analysis, with 60 of *Sparus aurata* and 60 of *Dicentrarchus labrax* for qualitative antibiotic analysis in Castro Bay (Italy).

Mantles from 54 *Sepia* spp. and 32 *Loligo* spp. were collected for cadmium analysis; stomachs and guts of 30 *Sparus aurata* and 20 *Dicentrarchus labrax* were sampled for microplastics analysis, with 60 of *Sparus aurata* and 60 of *Dicentrarchus labrax* for qualitative antibiotic analysis in Vlora Bay (Albania), per year.

Mantles of 80 samples of *Sepia* spp. and 80 of *Loligo* spp. were collected for cadmium analysis, stomachs and guts of 30 *Sparus aurata* and 30 *Dicentrarchus labrax* were sampled for microplastic analysis, and 60 of *Sparus aurata* and 60 of *Dicentrarchus labrax* were used for qualitative antibiotic analysis in Boka Kotorska Bay (Montenegro), per year.

Moreover, during the years 2021–2023, 100 samples of *Sparus aurata* and *Dicentrarchus labrax* were collected from each bay, to perform the next scientific evaluation of antibiotics using multi residual analysis. The quantitative analysis was carried out at the Department of Veterinary Medicine and Animal Science, University of Milan.

Samples intended for the analysis of cadmium and antibiotics were stored in sterile plastic containers. In contrast, samples intended for the analysis of microplastics were stored in plastic-free containers.

All samples were stored at −20 °C until analysis.

The fishermen selected for the project were first trained in order to collect samples of the same sizes in terms of length and weight to minimize the differences relating to the bioaccumulation/biomagnification of certain contaminants.

### 2.2. Cadmium Analysis in Digestive Glands and Mantles of Sepia *spp.* and Loligo *spp.*

#### 2.2.1. Digestion and Analysis

The mantles and glands of *Loligo* spp. and *Sepia* spp. were thawed and oven-dried at 105 °C for 24 h to a constant weight. Subsequently, acid digestion of the mantle and gland of fish samples followed standard methods [21,22,23]. They were dried at 105 °C for 4 h and grounded into a fine powder using a mortar and pestle. Then, 0.5 g of each ground sample was placed into a borosilicate beaker, and 12 mL of aqua regia (3:1 HCl/HNO_3_) was added. The beakers were covered with watch glasses and left for 16 h at room temperature. The samples were heated for 2 h at 80 °C. During heating, the watch glasses were removed and small amounts of 1% *v*/*v* HNO_3_ were periodically added to avoid drying of the samples. After the cooling of samples, they were filtered through Whatman 41 filter papers. All samples were made up to 50 mL with deionized water.

#### 2.2.2. Analytical Method

Fish samples were analyzed for Cd with the inductively coupled plasma mass spectrometer (Agilent 7500ce, Agilent Technologies, Santa Clara, CA, USA) equipped with a Cetac ASX-510 auto-sampler (Thermo Fisher Scientific, San Jose, CA, USA). To produce calibration curves from which the cadmium concentration was read out and to calibrate the instrument after appropriate dilutions, a standard heavy metal (Cd) solution (1000 mg/L) was employed; 1% (*v*/*v*) HNO_3_ was employed to prepare standard and blank solutions.

#### 2.2.3. Quality Control

The preparation of solutions and analysis were performed in a clean laboratory environment. The glassware was washed thoroughly with distilled water and detergent and dried in an oven. The reagents used for the analysis were of analytical grade and of high purity. Quality control, expressed as trueness, considered the following parameters based on Commission Regulation (EC) No 333/2007 (EU, 2007) [24]: limit of detection (LOD), defined as three times the standard deviation (SD) of the noise from six different sample blanks; limit of quantification (LOQ), defined as ten times; the SD of the noise from six different sample blanks; repeatability, in terms of the relative standard deviation (RSD) of measurements made on twelve blank samples spiked with 1 mg/kg of Cd, respectively. Uncertainty, expressed as expanded measurement uncertainty (U,) was evaluated based on repeatability data; unless otherwise stated, results are reported as the concentration ± U. Recovery was not calculated, due to the lack of extraction procedures, as stated by the above-mentioned regulation [24].

#### 2.2.4. Statistical Analysis

The descriptive statistics were performed using Microsoft Excel^®^ 2019 v17.0.

### 2.3. Microplastics Analysis

#### 2.3.1. Sample Collection and Preparation

Fish (*Sparus aurata* and *Dicentrarchus labrax*) were defrosted at room temperature. The whole gastrointestinal systems of fish species were removed from the anal orifice to the head area. The detection of microplastics was carried out on guts and stomachs of *Sparus aurata* and *Dicentrarchus labrax*. To avoid contamination with microplastics due to transportation or other environmental factors, the collected samples were stored in plastic-free containers and brought to the laboratory in a frozen condition at −20 °C, where they were analyzed. Three replicates per sample were carried out.

#### 2.3.2. Quality Control

All fluids (distilled water, saline solution, and hydrogen peroxide) were filtered with a cellulose nitrate filter membrane with a pore size of 1 μm and a diameter of 47 mm (Axiva Sichem Biotech, Delhi, India) in order to avoid contamination. Moreover, containers and beakers were washed three times with filtered distilled water before and after use and covered with aluminum foil to fend off airborne microplastics.

A blank extraction sample without fish samples was used to correct for any procedural contamination.

#### 2.3.3. Hydrogen Peroxide Treatment

A total of 200 g of the guts or stomachs was placed in a 1 L glass bottle, and 200 mL of 30% H_2_O_2_ (1:20 *w*/*v*) (Honeywell/Fluka, Charlotte, NC, USA) was added to digest the samples. For each stomach and gut sample, 3 replicates were prepared. The bottles were covered with aluminum foil and placed in an oscillation incubator at 65 °C, with 80 rpm for 24 h, and after that at room temperature for 24 or 48 h, depending on the complete digestion of the organic matter [25].

#### 2.3.4. Floatation and Filtration

To separate the microplastics from the digested samples via flotation, 800 mL of a filtered (supersaturated) NaCl solution (1.2 g mL^−1^) was added to each bottle containing the digested samples, mixed, and incubated overnight at room temperature. The aqueous phase (supernatant) was filtered through a cellulose nitrate membrane filter with a 5 μm pore size and 47 mm diameter (Axiva Sichem Biotech, Delhi, India) using Membrane–Laborpunpe (KNF Flodos AG, Sursee, Switzerland) under a vacuum system. Several sequential steps were carried out to maximize the recovery of MPs. The filters were placed in glass Petri dishes, covered, and dried at room temperature [25].

#### 2.3.5. Observation of the Filters and Detection of the Items

A stereomicroscope (Nikon, Calenzano, Italy) was used to observe the filters for potential plastic particles, and images were captured using a digital camera (Nikon X_Entry, Tokyo, Japan).

### 2.4. Antibiotics Analysis

During the first scientific evaluation (2018–2020), an antibiotic qualitative analysis was carried out using Premi^®^Test 100 (R-Biopharm, AG, Darmstadt, Germany) for *Sparus aurata* and *Dicentrarchus labrax* following the manufacturer’s instructions. Subsequently, a subsequent scientific evaluation of antibiotics using multi residual analysis was carried out (2021–2023).

#### 2.4.1. Multi Residual Analysis

Multi residual analysis was carried out following the protocol described by Chiesa L.M. et al., 2018 [26]. Briefly, 1 g of sample was spiked at 2 ng mL^−1^ with the IS and added to 100 µL of 20% TCA and 5 mL McIlvaine buffer (pH 4.0), for protein precipitation and extraction, respectively. After vortexing, sonication for 15 min, and centrifugation (2500× *g*, 4 °C, 10 min), the supernatant was defatted with 2 × 3 mL n-hexane. The extract was then purified using SPE Oasis HLB cartridges, and the eluate was evaporated and reconstituted in 200 µL of methanol:water (10:90 *v*/*v*) in a vial, ready to be analyzed via the injection of 10 µL.

#### 2.4.2. Chemicals and Reagents

The following solvents, reagents, and antimicrobial agent analytical standards were purchased from Merck (Darmstadt, Germany):

Methanol and n-hexane (hypergrade for LC-MS LiChrosolv^®^);
Trichloroacetic acid (TCA) crystals, disodium hydrogen phosphate dihydrate, citric acid monohydrate, and EDTA (for preparing EDTA-McIlvaine buffer solution, pH 4);
Ampicillin, penicillin G, cloxacillin, amoxicillin, penicillin V, oxacillin, dicloxacillin, nafcillin, enrofloxacin, ciprofloxacin, danofloxacin, marbofloxacin, flumequine, tetracycline, 4-epitetracycline, oxytetracycline, 4-epioxytetracycline, chlortetracycline, 4-epichloretracycline, doxycycline, sulfadiazine, sulfathiazole, sulfadimethoxine, sulfadimidine, and enrofloxacin d5 as the internal standards (IS).

Formic acid (98–100%) was provided from Riedel-de Haën (Sigma-Aldrich, St. Louis, MO, USA).

Water was purified using a Milli-Q system (Millipore, Merck KGaA, Darmstadt, Germany).

The Oasis HLB cartridges (3 mL, 60 mg) were supplied by Waters (Milford, MA, USA).

#### 2.4.3. Standard Solutions

Analytical standard stock solutions (1 mg mL^−1^) were prepared in methanol and kept at −20 °C; working solutions, at 10 and 100 ng mL^−1^, were prepared daily by diluting stock solution in methanol and were maintained at 4 °C.

#### 2.4.4. Sample Extraction

The selected samples were homogenized, and an aliquot of 1 g was spiked with the IS at a final 2 ng mL^−1^ and then were added to 100 µL of 20%. TCA and 5 mL McIlvaine buffer (pH 4.0) were used for, respectively, protein precipitation and extraction of the solvent.

After the vortexing, sonication for 15 min, and centrifugation (2500× *g*, 4 °C, 10 min), the supernatant was recovered, transferred to a clean centrifuge tube, and defatted with 2 × 3 mL n-hexane.

The final extracts were loaded and purified using SPE Oasis HLB cartridges under a vacuum, previously preconditioned with 3 mL of methanol and 3 mL of Milli-Q water, washed with 2 × 3 mL of methanol:water (5:95 *v*/*v*), and were eluted with 5 mL off methanol.

The eluate was evaporated in a rotary vacuum evaporator at 40 °C. The dried extract was resuspended in 200 µL of methanol:water (10:90 *v*/*v*), and transferred to an auto-sampler vial.

#### 2.4.5. HPLC-HRMS Analyses

An UPLC-HRMS system made of a Vanquish (Thermo Fisher Scientific, Waltham, MA, USA) coupled to a Thermo Orbitrap™ Exploris 120 (Thermo Fisher Scientific, Waltham, MA, USA), using a heated electrospray ionization (HESI) source, equipped with A Raptor ARC-18 5 µm, 120 × 2.1 mm column (Restek, Bellefonte, PA, USA), was used for the multi residual analysis of antibiotics in fish samples.

The mobile phase consisted of phase A (aqueous formic acid 0.1%) and B (methanol).

The run was performed at 0.3 mL min^−1^, and the injection volume was 10 µL.

The gradient started with 98% A (3 min), which was then increased linearly to 95% (at 10 min) and remained constant for 3 min; at the 14th min, the initial conditions were reached and with an equilibration time of 6 min.

Regarding detector parameters, the capillary and vaporizer temperatures were set at 330 and 280 °C, respectively, and then, the sheath and auxiliary gas were set at 35 and 15 arbitrary units (AUs) and the electrospray voltage was established at 3.50 and 3.0 kV, for positive and negative mode, respectively.

The full scan (FS) acquisition was mingled with a parallel reaction monitoring (PRM) mode, based on an inclusion list, to obtain the confirmatory response; the FS parameters were a resolution of 60,000 FWHM, a scan range of 125–1000 *m*/*z*, a standard automatic gain control (AGC), an RF lens % of 70, and an automatic maximum injection time.

The PRM parameters for the acquisition were 15,000 FWHM, with a standard AGC target, an automatic maximum injection time, and an isolation window of 1 *m*/*z*.

Fragmentation of the precursors was optimized with a two-step normalized collision energy (25 and 40 eV).

XcaliburTM 4.5 (Thermo Fisher Scientific, Waltham, MA, USA) was the software used.

#### 2.4.6. Method Validation

After the identification of samples in which we checked the absence of antibiotics, through preliminary screening, the method was validated to assess specificity, selectivity, precision, recovery, ruggedness, linearity, and the decision limit CCα, according to the Commission implementing Regulation (EU) 2021/808 [27]. A combination of experiments was carried out during 3 different days. The selectivity and specificity were carried out by analyzing 20 different blank samples, and that analysis was performed on 3 different days. Spiked blank samples were used to build calibration curves based on 5 points in triplicate. For the precision and recovery evaluation, six analysis replicates of 3 levels of concentrations (0.5 MRL, MRL, and 1.5 MRL; for substances without, MRL the minimum instrumental method was considered) were performed on each of the 3 days. Recovery was determined by comparing the fortified blank samples before extraction with those after extraction. The matrix effect was also calculated by comparing the peak areas of analytes spiked after extraction of a blank sample to the peak areas of standards in a neat solution mix, expressed as a percentage.

## 3. Results

### 3.1. Cadmium in Loligo *spp.* and Sepia *spp.*

*Loligo* spp. and *Sepia* spp. were analyzed for the presence of Cd for which the EU set MLs [28]. Table 1, Table 2 and Table 3 summarize the data (mg/kg wet weight) as the mean ± SD of Castro Bay, Vlora bay, Boka Kotorska bay, respectively. Trueness ranged from 102.3 to 105.3%. The LODs were 0.001 mg/kg for Cd. The LOQ was 0.01 mg/kg. Repeatability, expressed as RSDs, ranged from 2.5 to 6%. Uncertainty, related to a 1.0 mg/kg spike in cadmium in 12 blank samples, was between 0.05 and 0.11 mg/kg. Cd was detected in all samples from the three different bays (Table 1, Table 2 and Table 3). The Cd concentration in the gland was assessed only in Castro Bay and ranged between 1.2 mg/kg and 1.5 mg/kg in *Loligo* spp. and *Sepia* spp., respectively. The Cd concentration in the mantles of *Loligo* spp. and *Sepia* spp. ranged between 0.02 mg/kg up to 0.12 mg/kg in the three bays. All concentrations were lower than the ML set by (EU) 2023/915 [28], of 1 mg/kg, except from glands sampled in Castro Bay (1.2 and 1.5 ± 0.008 mg/kg).

### 3.2. Microplastics in Sparus aurata and Dicentrarchus labrax

All analyzed samples from the three different bays tested positive for the presence of microplastics in the stomach and gut of *Sparus aurata* and *Dicentrarchus labrax* (Table 4, Table 5 and Table 6). Four types of MPs were detected in our study: fibers, fragments, plastic films, and spherical granules. The contamination from airborne-microplastics in the blank samples was low, with an average value of 0.5 ± 0.20 compared to the maximum value of *Sparus aurata* samples from Castro Bay (6 ± 0.24 MPs/individual). Similarly, an average value of 0.3 ± 0.32 and 0.2 ± 0.13 of contamination from airborne-microplastic in the blank samples from Vlora Bay and Boka Kotorska Bay, respectively, was detected. Microplastics found in the blank samples were eliminated from the total count.

The lowest values were found in Boka Kotorska and Vlora bay, where about 2 MPs for each gut of *Sparus aurata* and *Dicentrarchus labrax* were detected, respectively.

Instead, the highest value was observed in stomachs of *Sparus aurata* from Castro Bay with an average value of 6 MPs/individual (Table 4, Table 5 and Table 6).

#### Statistical Analysis Results

Data obtained with the *t*-test showed that the number of microplastics in the samples was significantly higher than in the procedural blanks (*p* < 0.001). A significant difference between the number of MPs in the samples (*p* < 0.05) was revealed by the statistical analysis (ANOVA).

No significant difference was found between the results of the three replicates (*p* > 0.05).

### 3.3. Antibiotics in Sparus aurata and Dicentrarcus labrax

Quinolone and Tetracyclines were detected in 60 *Sparus aurata* and *Dicentrarchus labrax* samples from Castro Bay samples No antibiotic residues were found in *Sparus aurata* and *Dicentrarcus labrax* sampled in Vlora bay and Boka Kotorska bay (Table 7, Table 8 and Table 9).

#### 3.3.1. Results of Multiresidual Analysis

Multiresidual analysis confirmed the results obtained with the qualitative antibiotic analysis of *Sparus aurata* and *Dicentrarchus labrax* from the three different bays sampled during the 2018–2021 period. Antibiotics (Flumequiine, Tetracycline, Oxytetracycline, Doxycycline, Chlortetracycline) were found only in *Sparus aurata* and *Dicentrarchus labrax* samples from Castro Bay, with average values from 20.34 ± 0.56 up to 90.37 ± 0.36 ng g^−1^ (Table 10).

#### 3.3.2. Validation Performances

The selectivity of the method, assessed by injecting blank samples, did not show any interference where the analytes eluted. The selectivity also showed a good compliance with the relative RTs for each analyte, which were found to be within 2.5% tolerance, when compared with the standards, with peaks having a signal-to-noise ratio > 3. The mean recoveries for all analytes ranged between 90 and 113%. The matrix validation curves also demonstrated a good fit for all analytes, with correlation coefficients > 0.99. The intra- and inter-day repeatability values, which were calculated using one-way analysis of variance and expressed as coefficients of variation, were below 14 and 20%, respectively. The CCα values were from 1.58 to 601.60 ng g^−1^ wet weight (Table 11), on the basis of the presence of LMRs for the authorized substances.

Also, the method ruggedness was good in the considered matrices. A modest matrix effect was found, with values ranging from 90 to 112% for the various compounds in the fish samples.

## 4. Discussion

In the present study, the presence of cadmium, microplastics, and antibiotic was investigated in fish and cephalopods sampled in the three fishing areas selected for the EU Interreg Italy-Albania-Montenegro Project “ADRINET_244”.

Cadmium revealed values ranging from 0.02 mg/kg up to 0.12 mg/kg in the mantles of cephalopods (*Sepia* spp. and *Loligo* spp.) sampled in three bays, while an average value of 1.5 mg/kg was found in the digestive glands of *Sepia* spp. sampled in Castro Bay. The high value obtained for digestive glands of *Sepia* spp. confirms the primary role of these organs in the bioaccumulation and detoxification processes of cadmium. Contextually, the presence of this metal in the sampling area of Castro Bay suggests that it is also widespread in the other two bays considered in the ADRINET project. Furthermore, as described by previous studies [29,30], the cadmium values detected in the mantles of cephalopods reached values lower than the limit of 1 mg/kg allowed by the Commission Regulation (EU) No 915/2003, establishing a maximum level of contaminants in foodstuffs, fixing the specific limit for cadmium in cephalopods without viscera [28]. The higher accumulation of Cd in the digestive glands of *Sepia* spp. than in mantles could reflect the real Cd exposure of fishes, in particular cephalopods. In fact, it is well known that the content of Cd in the glands of cephalopods reflects its environmental availability, as reported by Raimundo et al., 2005 [31].

Regarding microplastics, our results indicate that both fish species analyzed (*Sparus aurata* and *Dicentrarchus labrax*) are vulnerable to microplastic ingestion. All analyzed samples from the three different bays tested positive for the presence of microplastics in the stomach and gut (Table 4, Table 5 and Table 6). Instead, the highest value was observed in the stomach of *Sparus aurata* from Castro Bay with an average value of 6 MPs/individual (Table 4, Table 5 and Table 6). These data could be caused by the fact that Castro is the most anthropized bay among the three areas considered in this study.

The lowest values were found in Boka Kotorska and Vlora bay, where about 2 MPs for each gut of *Sparus aurata* and *Dicentrarchus labrax* were detected, respectively.

However, there were no significant differences in the occurrence of microplastics between the two fish species.

Average values of ingested microplastics/individuals in *Sparus aurata* and *Dicentrachus labrax* detected in our study are higher than the number of microplastics/individuals observed by Mistri et al., 2022, in other Mediterranean fish species [32].

Microplastics, particularly those ingested by seafood and therefore transferable to humans, are a problem of a great concern since there is a lack of currently available knowledge on their effects in fish and consequently in humans through the food chain [32]. Nowadays, based on the lack of European regulations about microplastics in retailed seafood, our results suggest that they are required to fix the maximum limits of microplastics in seafood for human consumption, also considering their chemical characterization. In our study, we only detected microplastics in seafood without a chemical characterization since in our laboratories, there is no equipment useful for this analysis (e.g., micro-FTIR).

At least, the analysis of six different antibiotic types in *Sparus aurata* and *Dicentrarchus labrax* sampled in Castro Bay detected the presence of only two antibiotics classes, such as Tetracyclines and Quinolone. These results have scientific relevance because the antibiotic detection in the edible tissues of caught fish highlights the persistent aquatic and environmental pollution. Through the next multiresidual analysis of antibiotics carried out during the sampling period of 2021–2023 in the three different bays, it was possible to define and quantify antibiotic residues (Flumequiine, Tetracycline, Oxytetracycline, Doxycycline, Chlortetracycline) only in *Sparus aurata* and *Dicentrarchus labrax* samples from Castro Bay with average values from 20.34 ± 0.56 up to 90.37 ± 0.36 ng g^−1^ (Table 11). It is estimated that the presence of these antibiotic residues in fish sampled in Castro Bay could be caused by their employment in aquaculture, which is one of the activities of principal commercial interest in this area. However, according to Amalgesin et al., is well-known that antibiotics, such as Tetracyclines, are used in fish farms [33]. Moreover, the CCα values were from 1.58 to 601.60 ng g^−1^ wet weight (Table 10), on the basis of the presence of LMRs for the authorized substances, and they were similar to or lower than other studies on this topic [34,35].

These results are in compliance with the EU Reg. 37/2010 on “pharmacologically active substances and their classification regarding maximum residue limits in foodstuffs of animal origin” [36,37]. However, the detection of a low levels of antibiotic residues represents a risk for living organisms and natural ecosystems since they could cause and spread antibiotic resistance. Based on recent studies, the increased accumulation of antibiotics in fish may also be due to the presence of microplastics [38]. Therefore, monitoring and cleaning campaigns are necessary both to understand where these pollutions come from and to assess the possible risk for public health. Moreover, based on the implementation of the ADRINET project in the three sub-regions analyzed in our study, this scientific approach could be adopted at a regional/national level to monitor the seawater pollution and, consequently, seafood safety.

## 5. Conclusions

The low average values of the contaminants investigated in our study in caught fish highlight that environmental conditions of the three bays analyzed by the ADRINET project partners (Italy, Albania, Montenegro) are positive and not affected by intensive anthropogenic activity. However, the most alarming data include the presence of microplastics dispersed in these bays and then found in high values in the gut and stomach of some fishery products.

Moreover, the ADRINET project was created with the aim to promote the international cooperation for territorial development and to improve the quality of life of the populations included in the project. For this reason, data obtained in our study will allow for increasing knowledge about the contamination of the bays investigated and to promote strategies that guarantee the quality and safety of seafood for the economic growth in the areas investigated.

## Figures and Tables

**Table 1:** Cadmium in glands and mantles of *Sepia* spp. and *Loligo* spp. sampled in Castro Bay.

Number of Samples	Sample Types	Parameters	Unit of Measures	Mean ± SD *	Maximum Limit Allowed in Flesh
80	Glands *Sepia* spp.	Cd	mg/kg	1.5 ± 0.008	1.0
80	Glands *Loligo* spp.	Cd	mg/kg	1.2 ± 0.008	1.0
80	Mantles *Sepia* spp.	Cd	mg/kg	0.02 ± 0.008	1.0
80	Mantles *Loligo* spp.	Cd	mg/kg	0.8 ± 0.008	1.0

* Concentrations (mg/kg), expressed as means ± SDs of cadmium (Cd) in in glands and mantles of *Sepia* spp. and *Loligo* spp. Means and SDs were calculated only based on positive samples.

**Table 2:** Cadmium in mantles of *Sepia* spp. and *Loligo* spp. sampled in Vlora Bay.

Number of Samples	Sample Types	Parameters	Unit of Measure	Mean ± SD *	Maximum Allowed Limit in Flesh
54	Mantles *Sepia* spp.	Cd	mg/kg	0.02 ± 0.008	1.0
32	Mantles *Loligo* spp.	Cd	mg/kg	0.12 ± 0.008	1.0

* Concentrations (mg/kg), expressed as means ± SDs of cadmium (Cd) in mantles of *Sepia* spp. and *Loligo* spp. Means and SDs were calculated only based on positive samples.

**Table 3:** Cadmium in mantles of *Sepia* spp. and *Loligo* spp. sampled in Boka Kotorska Bay.

Number of Samples	Sample Types	Parameters	Unit of Measure	Mean ± SD *	Maximum Allowed Limit in Flesh
80	Mantles *Sepia* spp.	Cd	mg/kg	0.08 ± 0.008	1.0
80	Mantles *Loligo* spp.	Cd	mg/kg	0.11 ± 0.008	1.0

* Concentrations (mg/kg), expressed as means ± SDs of cadmium (Cd) in mantles of *Sepia* spp. and *Loligo* spp. Means and SDs were calculated only based on positive samples.

**Table 4:** Microplastic in Sparus aurata and Dicentrarcus labrax from Castro Bay.

Species		Nr. of Analyzed Samples	Microplastics *	
			Stomach	Gut
Species selected for study by ADRINET	*Spaurus* *aurata*	90	6 ± 0.24	3 ± 0.3
	*Dicentrarchus labrax*	70	4 ± 0.28	3 ± 0.7

* Average values of MPs/individuals (200 g) for *Sparus aurata* and *Dicentrarchus labrax*.

**Table 5:** Microplastics in *Sparus aurata* and *Dicentarchus labrax* from Vlora Bay.

Species		Nr. of Analyzed Samples	Microplastics *	
			Stomach	Gut
Species selected for study by ADRINET	*Spaurus* *aurata*	30	4 ± 0.56	2 ± 0.29
	*Dicentrarchus labrax*	20	3 ± 0.47	2 ± 0.16

* Average values of MPs/individuals (200 g) for *Sparus aurata* and *Dicentrarchus labrax*.

**Table 6:** Microplastics in *Sparus aurata* and *Dicentarchus labrax* from Boka Kotorska bay.

Species		Nr. of Analyzed Samples	Microplastics *	
			Stomach	Gut
Species selected for study by ADRINET	*Spaurus* *aurata*	30	3 ± 0.18	2 ± 0.23
	*Dicentrarchus labrax*	30	4 ± 0.43	2 ± 0.12

* Average values of MPs/individuals (200 g) for *Sparus aurata* and *Dicentrarchus labrax*.

**Table 7:** Antibiotics in *Sparus aurata* and *Dicentrarcus labrax* from Castro Bay.

Nr. of Samples	Sample Types	Antibiotics	Results
60	*Spaurus* *aurata*	Thiamphenicol	Not found
		Streptomycin	not found
		Tylosin	not found
		Quinolone	Positive
		Ceftiofur	not found
		Tetracyclines	Positive
60	*Dicentrarchus labrax*	Thiamphenicol	not found
		Streptomycin	not found
		Tylosin	not found
		Quinolone	Positive
		Ceftiofur	not found
		Tetracyclines	Positive

**Table 8:** Antibiotics in *Sparus aurata* and *Dicentrarcus labrax* spp. from Vlora Bay.

Nr. of Samples	Sample Types	Substance (Antibiotics)	Results
60	*Spaurus* *aurata*	Thiamphenicol	not found
		Streptomycin	not found
		Tylosin	not found
		Quinolone	not found
		Ceftiofur	not found
		Tetracyclines	not found
60	*Dicentrarchus labrax*	Thiamphenicol	not found
		Streptomycin	not found
		Tylosin	not found
		Quinolone	not found
		Ceftiofur	not found
		Tetracyclines	not found

**Table 9:** Antibiotics in *Sparus aurata* and *Dicentrarcus labrax* spp. from Boka Kotorska bay.

Nr. of Samples	Sample Types	Substance (Antibiotics)	Results
60	*Spaurus aurata*	Thiamphenicol	not found
		Streptomycin	not found
		Tylosin	not found
		Quinolone	not found
		Ceftiofur	not found
		Tetracyclines	not found
60	*Dicentrarchus labrax*	Thiamphenicol	not found
		Streptomycin	not found
		Tylosin	not found
		Quinolone	not found
		Ceftiofur	not found
		Tetracyclines	not found

**Table 10:** List of detected antibiotics using multi residual analysis of antibiotics in *Sparus aurata* and *Dicentrarchus labrax* sampled in the three different bays of ADRINET.

Nr. of Samples	Sample Type	Substance (Antibiotics)	Unit of Measure	Results MD ± SD *		
				Castro Bay	Vlora Bay	Boka Kotorska Bay
100	*Spaurus aurata*	Flumequiine	ng g^−1^	20.34 ± 0.56	Not found	Not found
		Tetracycline	ng g^−1^	60.45 ± 0.34	Not found	Not found
		*Oxytetracycline*	ng g^−1^	60 ± 0.37	Not found	Not found
		Doxycycline	ng g^−1^	87.37 ± 0.52	Not found	Not found
		Chlortetracycline	ng g^−1^	78.20 ± 0.51	Not found	Not found
100	*Dicentrarchus labrax*	Flumequiine	ng g^−1^	24.43 ± 0.12	Not found	Not found
		Tetracycline:	ng g^−1^	56.72 ± 0.23	Not found	Not found
		*Oxytetracycline*	ng g^−1^	54 ± 0.21	Not found	Not found
		Doxycycline	ng g^−1^	90.37 ± 0.36	Not found	Not found
		Chlortetracycline	ng g^−1^	81.76 ± 0.18	Not found	Not found

* Average values expressed as the MD ± SD. Means and SDs were calculated only based on positive samples.

**Table 11:** Validation parameters for all antibiotics.

Analyte	MRL (ng g^−1^)	Spiked Levels (ng g^−1^)	CCα (ng g^−1^)	Recovery * (%) (*n* = 18)	Repeatability *		
					Intra-Day (CV; *n* = 6)	Inter-Day (CV; *n* = 18)	Matrix Effect %
		25.00		100	14	20	
Amoxicillin	50	50.00	50.20	92	9	16	90
		75.00		101	8	10	
		25.00		90	14	20	
Ampicillin	50	50.00	50.55	98	13	14	93
		75.00		100	9	9	
		150.00		95	14	17	
Cloxacillin	300	300.00	301.42	97	11	13	92
		450.00		98	9	10	
		150.00		93	13	18	
Dicloxacillin	300	300.00	301.16	97	12	17	91
		450.00		99	11	11	
		25.00		90	14	19	
Penicillin G	50	50.00	50.30	92	13	17	95
		75.00		93	13	14	
		1.25		90	14	20	
Penicillin V	/	2.50	2.50 **	90	14	18	92
		3.75		92	12	13	
		150.00		92	13	17	
Oxacillin	300	300.00	300.16	95	11	15	94
		450.00		95	9	11	
		2.50		102	14	20	
Nafcillin	/	5.00	5.00 **	97	13	20	94
		7.50		101	13	18	
		50.00		95	14	16	
Ciprofloxacin	100	100.00	101.37	105	14	16	101
		150.00		98	11	12	
		50.00		100	8	15	
Enrofloxacin	100	100.00	101.20	100	8	15	103
		150.00		100	7	8	
		50.00		97	14	20	
Danofloxacin	100	100.00	100.35	103	13	20	105
		150.00		98	13	18	
		0.70		103	14	20	
Marbofloxacin	/	1.50	1.58 **	97	14	15	107
		2.25		101	8	10	
		300.00		99	13	17	
Flumequine	600	600.00	601.60	99	11	15	100
		900.00		91	9	11	
		50.00		92	7	11	
Chlortetracycline	100	100.00	100.62	103	5	11	102
		150.00		98	7	10	
		50.00		104	14	20	
Doxycycline	100	100.00	100.30	96	13	20	112
		150.00		101	12	13	
		50.00		102	10	16	
Oxytetracycline	100	100.00	101.00	98	8	15	104
		150.00		101	9	9	
		50.00		99	14	20	
Tetracycline	100	100.00	101.10	113	10	12	103
		150.00		96	9	10	
		50.00		96	14	20	
Sulphathiazole	100	100.00	101.55	96	10	17	102
		150.00		99	9	11	
		50.00		101	8	11	
Sulphadimidine	100	100.00	100.73	99	7	9	101
		150.00		100	7	7	
		50.00		102	11	18	
Sulphadiazine	100	100.00	100.80	102	9	15	105
		150.00		104	9	11	
		50.00		97	12	19	
Sulphadimethoxine	100	100.00	101.23	99	11	13	108
		150.00		93	10	11	

* The three values are related to the three validation levels: 0.5 MRL, MRL, 1.5 MRL; ** the CCα for substances without MRL was calculated on the basis of the minimum instrumental level.

## Data Availability

The original contributions presented in the study are included in the article, and further inquiries can be directed to the corresponding author.
